# Rapid identification of *Bactrocera zonata* (Dip.: Tephritidae) using TaqMan real-time PCR assay

**DOI:** 10.1371/journal.pone.0205136

**Published:** 2018-10-04

**Authors:** Marzieh Koohkanzade, Mohammad Zakiaghl, Manpreet K. Dhami, Lida Fekrat, Hussein Sadeghi Namaghi

**Affiliations:** 1 Department of Plant Protection, Faculty of Agriculture, Ferdowsi University of Mashhad, Mashhad, Iran; 2 Biodiversity & Conservation, Manaaki Whenua Landcare Research, Lincoln, New Zealand; Sichuan University, CHINA

## Abstract

Tephritid fruit flies are ranked as one of the most damaging groups of insect pests. Morphological identification of fruit flies is mainly performed on adults due to the lack of adequate identification keys for immature stages. The peach fruit fly, *Bactrocera zonata* (Saunders), infests some of the principal commercial fruits and vegetables. It is, therefore important to avert its global dispersal, particularly by accurately identifying this species at ports of entry. In this study, a TaqMan real-time polymerase chain reaction (PCR) was developed for the accurate identification and sensitive detection of the peach fruit fly. A novel set of primers and probe were designed to specifically identify the mitochondrial cytochrome oxidase I (COI) gene. All specimens of peach fruit fly (including various life stages) were detected, and no cross reactivity with other tested tephritids were observed. Since this assay performed equally well with crushed insects and purified DNA, we note added efficiency by eliminating DNA extraction step. Considering the speed, specificity as well as sensitivity of the assay, Taqman real-time PCR can be used as a swift and specific method for pest species at ports of entry.

## Introduction

Accurate, rapid and reliable identification of insect pests is a crucial first step in implementing an efficacious management program. Tephritid fruit flies are ranked as one of the most damaging insect pests causing immense economic losses by rendering infected fruits unfit for human consumption [1]. The rejection of fruit cargos due to the presence of maggots is a substantial threat for agricultural and horticultural industries in any fruit-producing country [2]. Many countries either impose quarantine restrictions on fruit and vegetable imports from countries infested with specific fruit fly species or performed expensive treatments such as fumigation before importing fresh produce [3,4]. Despite severe quarantine procedures, tephritids, especially *Bactrocera* spp. (Macquart), continue to expand their global range, establishing in previously pest-free regions.

The genus *Bactrocera* is diverse, with highly polyphagous species, of which up to 50 are considered serious pests [3,4,5]. One such species, peach fruit fly, *Bactrocera zonata* (Saunders), is a notorious, destructive and widespread pest of fruits causing severe losses to fruit production globally.

Containing the spread of pest fruit flies is imminently important, especially with a sharp rise in global trade. A direct result of which is the high volume of suspected tephritids associated with goods in transit or in active orchard surveys, often as larval stages [6,7,8,9]. Accurate species identification from larval stages is not possible, with heavy reliance on risky and time-consuming procedures such as rearing adults [10,11]. The uncertainty of results and possibility of further economic loss compounds this problem, especially for border biosecurity. In recent years, molecular techniques have taken center stage, as rapid and accurate diagnostics become indispensable for border biosecurity. Polymerase chain reaction based methods such as DNA barcoding, restriction fragment length polymorphism are being used for the identification of various pests around the world [12,13,14,15,16,17]. Microsatellites are also being used for the identification of various insect pests including tephritid species [18,19,20]. Of these, real-time PCR techniques have emerged as the fastest and most sensitive diagnostic method, so far applied to various insect pests, including Thysanoptera [21,22], Lepidoptera [23,24], aphids [25,26] and also fruit flies [27,28,29]. While elimination of post-PCR electrophoresis and amplicon sequencing reduces assay time, it is the increased sensitivity of the TaqMan marker system and life-stage independence that make this method universally applicable [30,31].

In this study, we developed a TaqMan real-time PCR assay for *B*. *zonata* diagnosis that targeted the COI region. To determine the specificity of detection, a total of 19 species of fruit flies were tested with the developed assay. To assess the sensitivity of TaqMan real-time PCR, different concentrations of template DNA of *B*. *zonata* were used. Detection system was also evaluated using DNA isolated from single larvae, pupae and adults of *B*. *zonata*.

## Materials and methods

### Sample collection

*Bactrocera zonata* specimens were collected using McPhaill traps baited with Methyl Eugenol in July 2016 from Sistan and Balouchestan and Hormozgan provinces, southeastern Iran (Table 1). These samples were collected on private lands with express permissions of the landowners. No samples were collected in national parks, therefore collection permits were not required. For the specificity testing, the samples were provided by the Ministry for Primary Industries, New Zealand. No endangered or threatened flies were included in the study. Ethics approval was not required as insects are not classified as animals for the purposes of the Animal Welfare of Iran Legislation.

**Table 1:** List of species used in primer and probe design alignment.

Species	Origin	Coordinates	Life-stage	Accession number	Source
*B*. *zonata*	Konarak (Sistan and Baluchestan Province), Iran	25.4039° N, 60.3733° E	adult	MF419187	This study
*B*. *zonata*	Minab (Hormozgan Province), Iran	27.1372° N, 57.0675° E	adult	MF419188	This study
*B*. *zonata*	Chabahar (Sistan and Baluchestan Province), Iran	25.2969° N, 60.6459° E	adult	MF419189	This study
*B*. *zonata*	GenBank (Thailand)	-	-	KT595002	[32]
*B*. *zonata*	GenBank (India)	-	-	KJ476960	[33]
*B*. *cucurbitae*	China	-	adult	MF419190	This study
*B*. *cucurbitae*	GenBank (Japan)	-	-	AY530900	Unpublished
*B*. *dorsalis*	China	-	adult	MF419191	This study
*B*. *dorsalis*	Australia	-	adult	MF419192	This study
*B*. *dorsalis*	Austria	-	adult	MF419193	This study
*B*. *dorsalis*	GenBank (Nigeria)	-	-	AB972846	[9]
*B*. *tau*	China	-	adult	MF419194	This study
*B*. *tau*	GenBank (Oriental)	-	-	GQ458047	[34]
*Carpomyia vesuviana*	Ghaen (Khorasan-e-Jonoubi province), Iran	33.7227° N, 59.1788° E	adult	MF419195	This study
*C*. *vesuviana*	GenBank	-	-	HQ687210	Unpublished
*Ceratitis capitata*	Golestan province, Iran	-	adult	MF419196	This study
*C*. *capitata*	GenBank	-	-	JQ345482	[35]
*B*. *oleae*	Golestan province, Iran	-	adult	MF419197	This study
*B*. *latifrons*	Thailand	-	adult	MF419198	This study
*B*. *latifrons*	Thailand	-	adult	MF419199	This study
*B*. *latifrons*	GenBank	-	-	FJ903498	[36]
*Dacus ciliatus*	Neyshabour, Khorasan-e-Razavi province), Iran	36.2141° N, 58.7961° E	adult	-	This study
*D*. *ciliatus*	GenBank (Iran)	-	-	MF192740	Unpublished

### DNA extraction, PCR and sequencing

Total genomic DNA was extracted from the thorax of adult individuals, a 2–3 mm piece of the larval body or pupae using DNeasy Blood and Tissue Kit (Qiagen) according to manufacturer's instructions.

The Uea 7 and Uea 10 primers [37] were used for PCR amplification resulting in 680-bp long COI fragment. PCR amplification was performed in 25 μl volume containing 1 μl DNA template, 10.5 μl ddH2O, 12.5 μl of a PCR master mix (Amplicon) and 1μl of each of forward and reverse primers (10μmol/L). Negative controls were included in each PCR run. The thermocycling protocols included an initial denaturation of 94°C for 5 min, followed by 30 cycles of 94°C for 40 s, 51°C for 30 s and 72°C for 1 min, ending with a final extension step of 72°C for 10 min. PCR products were then electrophoresed on a 1% agarose gel. The size of DNA fragments was estimated by comparing to a 100 bp DNA ladder (Fermentas). A sterile razor blade was used to excise the bands corresponding to the target PCR products, and then purification was carried out with QIAquick gel extraction kit (Qiagen). Purified PCR amplicons were sent to Macrogen Corporation (Seoul, South Korea) for bidirectional sequencing. All tephritid species sequences were deposited in GenBank under the accession numbers provided in Table 1.

### TaqMan probe and real-time PCR primer design

COI gene sequences of tephritids obtained from samples collected in this study and additional sequences downloaded from GenBank were used in the assay design process (Table 1). GenScript Real-time PCR (TaqMan) Primer Design was used to design the primers and the probes. Comparisons of the designed primers and probes with locations in a COI alignment were then carried out to select combinations that would maximize discrimination between *B*. *zonata* and other examined tephritid species. The COI primers generated an amplicon of 100bp. The resulting primers and probe information is provided in Table 2.

**Table 2:** TaqMan probe and primers used in this study.

Name	Sequence	bp	Tm (°C)	Product (bp)
BzonF	AGCCACATTACATGGTACACAACT	24	52	
BzonR	AGGACAACTCCTGTTAATCCTCCT	24	52	100
BzonP	FAM-CTCCAGCTATACTGTGGGCCCTAGGA-TAMARA	26	63	

### Real-time PCR assay

All reactions were performed on an ABI7300 real-time PCR instrument. Gradients of temperatures (60–65°C) and primer concentrations (300 and 400 nM) were used in order to optimize the PCR conditions (Table 3). Reactions were carried out in duplicate with 25 μl final volume containing 2.5 μl of PCR buffer, 1 μl of each forward and reverse primers, 0.5 μl TaqMan probe, 16.5 μl ddH2O and 1μl template DNA. The real-time cycling parameters were as follows: an initial denaturation at 95°C for 30 s, followed by 40 cycles of denaturation at 94°C for 5 s and annealing and extension at 63°C for 30s (Table 3).

**Table 3:** Reaction components and cycling conditions of real-time PCR assay.

Reaction composition			Cycling conditions			
Component	Final concentration		Step	Temperature	Time	
	Simple	Duplex				
Primer F (BzonF)	400nM	400nM	Initial denaturation	95°C	30sec	
Primer R (BzonR)	400nM	400nM	Denaturation	94°C	5sec	×40 cycles
Probe (FAM) BzonP	200nM	200nM	Annealing/ extension	63°C	30sec	
18SF		50nM				
18SR		50nM				
18S probe (VIC)		50nM				
PCR buffer	2.5μl	2.5μl				
Taq polymerase	0.5	0.5				
dNTPs	0.5 μl	0.5 μl				
DNA template	1μl	1μl				
ddH2O	adjust volume to 25 μl					

### Specificity of Real-time PCR assay

A real-time PCR protocol was tested for specificity using a panel consisting of 19 non-target species obtained from 13 different countries around the world (Table 4). All of these control species were sympatric, closely related or have shared host plants with *B*. *zonata*. Prior to performing the real-time PCR, conventional PCR and sequencing were carried out for identifying all of the samples used for assay validation. The samples were also tested using a TaqMan 18S internal control real-time PCR (Applied Biosystems, USA) according to manufacturer`s instructions in the duplex format; i.e. *B*. *zonata* primers and probe along with 18S internal control primers and probe, to ascertain whether non-amplification was a result of non-target samples rather than insufficient quality/quantity of the DNA used in the assay or the existence of inhibitory compounds [24].

**Table 4:** List of fruit fly species used in the real-time PCR assays.

Sample	Accession number	Species	Life stage	Origin/Population	COI		18s internal control	
					Result	Mean Cq	Result	Mean Cq
1	MF419187	*B*. *zonata*	adult	Iran, Konarak (Sistan & Baluchestan Province) (25.4039° N, 60.3733° E)/ Population 1	+	13.3885	+	15.03
2	-	*B*. *zonata*	adult	Iran, Dehestan-e-Kahir (Sistan & Baluchestan Province) (25°35′16″N 60°07′42″E))	+	13.3989	+	13.33
3	MF419188	*B*. *zonata*	adult	Iran, Minab (Hormozgan Province) (27.1372° N, 57.0675° E)/ Population 2	+	13.4552	+	10.97
4	-	*B*. *zonata*	adult	Iran, Rudan (Hormozgan province) (27.4381° N, 57.1803° E)/ Population 3	+	13.5411	+	12.92
5	-	*B*. *zonata*	adult	Iran, Zarabad (Hormozgan province) (26°19′12″N 57°20′24″E))/ Population 4	+	13.4937	+	11.76
6	-	*B*. *zonata*	larva	Iran, Chabahar (Sistan and Baluchestan Province) (25.2969° N, 60.6459° E)	+	13.78	+	14.31
7	-	*B*. *zonata*	Larva (1^st^ instar)	Iran, Chabahar (Sistan and Baluchestan (25.2969° N, 60.6459° E)	+	13.45	+	14.32
8	-	*B*. *zonata*	Larva (3^rd^ instar)	Iran, Sistan and Baluchestan Province	+	13.36	+	13.20
9	-	*B*. *zonata*	pupa	Iran, Sistan and Baluchestan Province	+	13.46	+	12.60
10	-	*B*. *zonata*	adult	Iran, Tiskupan (Sistan & Baluchestan Province) (25.3218° N, 60.7548° E)/ Population 5	+	13.47	+	12.92
11	-	*B*. *zonata*	adult	Iran, Minab (Hormozgan Province) (27.1372° N, 57.0675° E)	+	14.10	+	10.92
12	MF419189	*B*. *zonata*	adult	Iran, Chabahar (Sistan and Baluchestan Province) (25.2969° N, 60.6459° E)/ Population 6	+	13.70	+	13.54
13	-	*B*. *zonata*	adult	Iran, Minab, (Hormozgan Province) (27.1372° N, 57.0675° E)	+	13.25	+	12.23
14	-	*B*. *zonata*	larva	India	+	12.3757	+	11.36
15	-	*B*. *zonata*	larval tissue	India	+	12.4122	+	11.64
16	-	*B*. *zonata*	egg	Unknown/ Collected from Guava	+	12.4616	+	11.64
17	KT864743	*B*. *carambolae*	larva	Indian apple	-	NA	+	10.02
18	KT864734	*B*. *carambolae*	adult leg	Unknown/ collected via border interception in New Zealand by Plant Health & Environment Laboratory	-	NA	+	18.12
19	KT864819	*B*. *carambolae*	adult	India	-	NA	+	19.56
20	-	*B*. *carambolae*	larva	Indonesia	-	NA	+	13.02
21	-	*B*. *carambolae*	larva	Indonesia	-	NA	+	15.56
22	KT864769	*B*. *tryoni*	adult abdomen	Brisbane, QLD, AU	-	NA	+	11.68
23	KT864775	*B*. *tryoni*	adult abdomen	Wodonga, Victoria, AU	-	NA	+	12.31
24	KT864721	*B*. *aquilonis*	adult abdomen	Broome, WA, AU	-	NA	+	13.18
25	KT864723	*B*. *aquilonis*	adult abdomen	Kunnunura, WA, AU	-	NA	+	15.05
26	KT864748	*B*. *invadens*	adult abdomen	Kenya	-	NA	+	12.29
27	KT864744	*B*. *invadens*	Pupa empty	India	-	NA	+	16.55
28	KT864735	*B*. *correcta*	adult abdomen	Sri Lanka	-	NA	+	12.04
29	-	*B*. *correcta*	adult	Yunnan Province, China	-	NA	+	15.06
30	KT864821	*B*. *kandiensis*	adult abdomen	Sri Lanka	-	NA	+	14.20
31	KT864822	*B*. *cucurbitae*	adult abdomen	Sri Lanka	-	NA	+	15.97
32	KT864823	*B*. *cucurbitae*	adult abdomen	Sri Lanka	-	NA	+	15.08
33	MF419190	*B*. *cucurbitae*	adult	China	-	NA	+	15.00
34	-	*B*. *dorsalis*	adult	Taiwan	-	NA	+	20.91
35	KT864737	*B*. *dorsalis*	adult	Vietnam	-	NA	+	12.47
36	-	*B*. *dorsalis complex*	papa	French Polynesia	-	NA	+	12.08
37	-	*B*. *dorsalis*	adult	Vietnam	-	NA	+	12.35
38	MF419191	*B*. *dorsalis*	adult	China	-	NA	+	10.74
39	MF419192	*B*. *dorsalis*	adult	Australia	-	NA	+	12.18
40	MF419193	*B*. *dorsalis*	adult	Austria	-	NA	+	10.74
41	-	*B*. *jarvisi*	adult	Hunter St, Broome, WA, AU	-	NA	+	13.65
42	-	*B*. *jarvisi*	adult	Broome (Trapsite25), WA, AU	-	NA	+	13.30
43	KT864798	*C*. *cosyra*	adult	Zambia	-	NA	+	16.34
44	KT864799	*C*. *rosa*	adult	South Africa	-	NA	+	12.24
45	KT864805	*D*. *pornia*	adult abdomen	Maroochy, QLD, AU	-	NA	+	10.95
46	KT864806	*D*. *pornia*	adult abdomen	Maroochy, QLD, AU	-	NA	+	10.86
47	KT864810	*D*. *pornia*	adult abdomen	Maroochy, QLD, AU	-	NA	+	11.73
48	KT864812	*D*. *pornia*	adult abdomen	Maroochy, QLD, AU	-	NA		11.63
49	-	*B*. *melanotus*	larva	Cook Islands	-	NA	+	16.94
50	KT864760	*B*. *papaye*	adult	Indonesia	-	NA	+	12.30
51	MF419194	*B*. *tau*	adult	China	-	NA	+	12.69
52	MF419198	*B*. *latifrons*	adult	Thailand	-	NA	+	18.94
53	MF419199	*B*. *latifrons*	adult	Thailand	-	NA	+	18.57
54	MF419197	*B*. *oleae*	adult	Iran, Golestan province	-	NA	+	14.68
55	MF419195	*Carpomyia vesuviana*	adult	Iran, Ghaen (Khorasan-e-Jonoubi province) (33.7227° N, 59.1788° E)	-	NA	+	21.06
56	MF419196	*C*. *capitata*	adult	Iran, Golestan province	-	NA	+	14.01

### In-silico testing for specificity

We downloaded up to five COI sequences from GenBank for an additional 34 species that have been reported to be closely related to *Bactrocera zonata* [38]. We aligned a total of 131 sequences including four *B*. *zonata* sequences to form the in-silico test set using Clustal W. Using the “Test with saved primers” tool in Geneious 10.1.3 (Biomatters Inc, New Zealand), we checked for non-specific primer/probe binding (allowing 1 mismatch in each) against this expanded set of species (S1 Appendix).

### Sensitivity evaluation, amplification efficiency, repeatability and reproducibility of the real-time PCR

Sensitivity of the selected TaqMan probe for *B*. *zonata* was evaluated in PCR runs with serial dilutions of *B*. *zonata* DNA with the same primer concentration. The estimation of DNA concentrations of samples were performed using a spectrophotometer (Thermo).

For each sample, a PCR assay was carried out that included 1 μl of template at the original sample concentration and then a dilution series (10^1^−10^3^) with each concentration in triplicate. The Cq values, the number of PCR cycles needed to detect DNA template in the reaction, for each dilution were plotted against logarithmic values of target copy numbers. The fewer the number of copies of the target DNA in the PCR template, the higher the Cq value.

The efficiency was calculated and converted to percentage efficiency utilizing the formulas E = (10 − 1/slope) [39] and E% = (E-1)×100 [11], respectively. Moreover, *r*^*2*^ was recorded as the fit of the slope. By utilizing standard curve data, performance indicators such as linear dynamic range and limit of detection were determined [40,41,27]. Percent coefficient of variation (%CV) was used to report the repeatability (intra-run variation) and reproducibility (inter-run variation) of the assay. To calculate % CV for each sample, six samples were examined in 4 replicates in two identical but separate runs and the resulting data were compared to make an estimation of reproducibility.

To further reduce assay time and increase assay convenience, we evaluated the sensitivity of this method on *B*. *zonata* samples without the DNA extraction step. For this test, one individual was crushed in 20 μl of sterile distilled water and then 1 μl of the homogenized solution was utilized as template for the real-time PCR reaction.

## Results

### Real-time PCR

**Fig 1:**
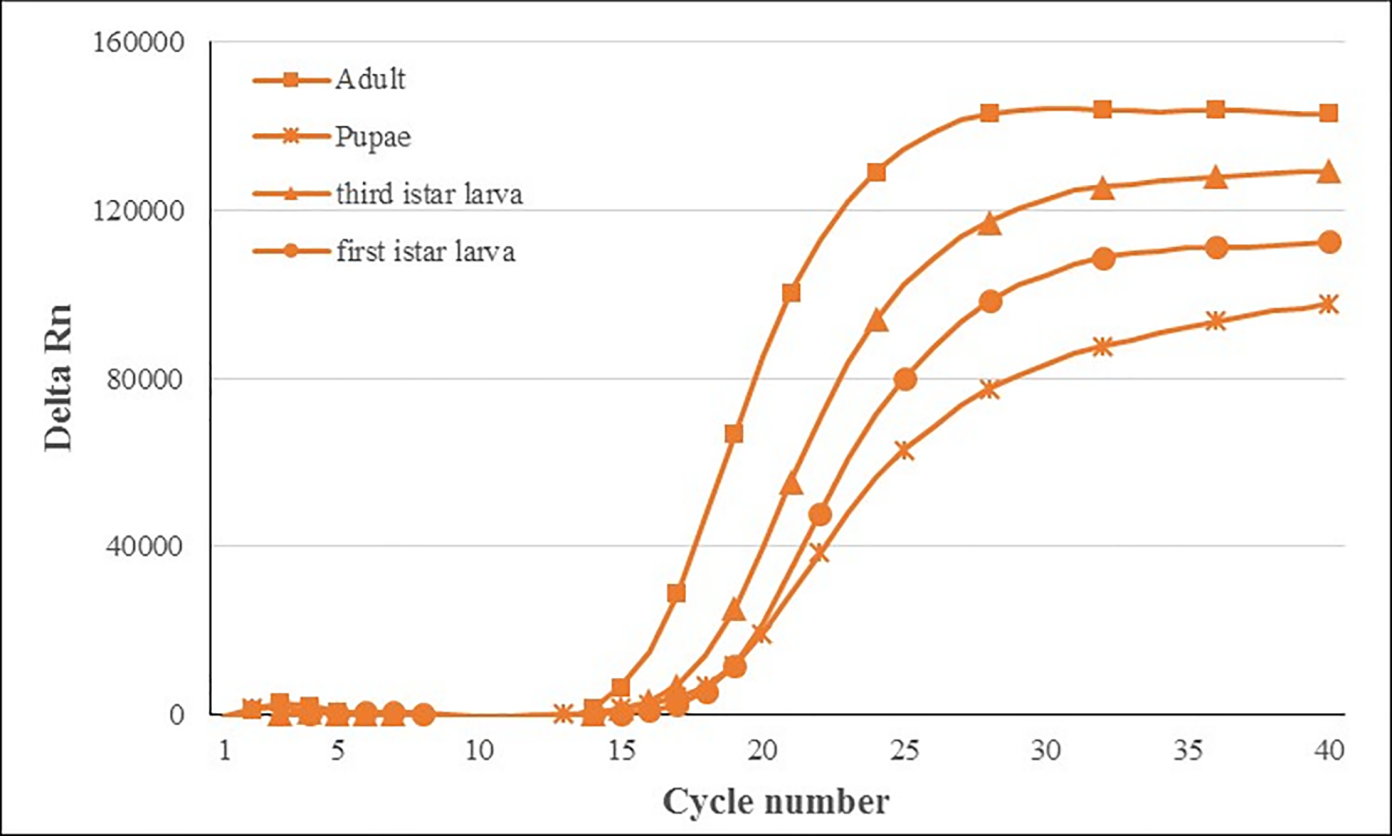
Amplification plot obtained during real-time PCR on DNA of various developmental stages of *B*. *zonata* (Population 6 in Table 4: Chabahar (Sistan and Baluchestan Province)) with optimized primer (400nM) and probe concentrations (200nM). The threshold is set automatically.

**Fig 2:**
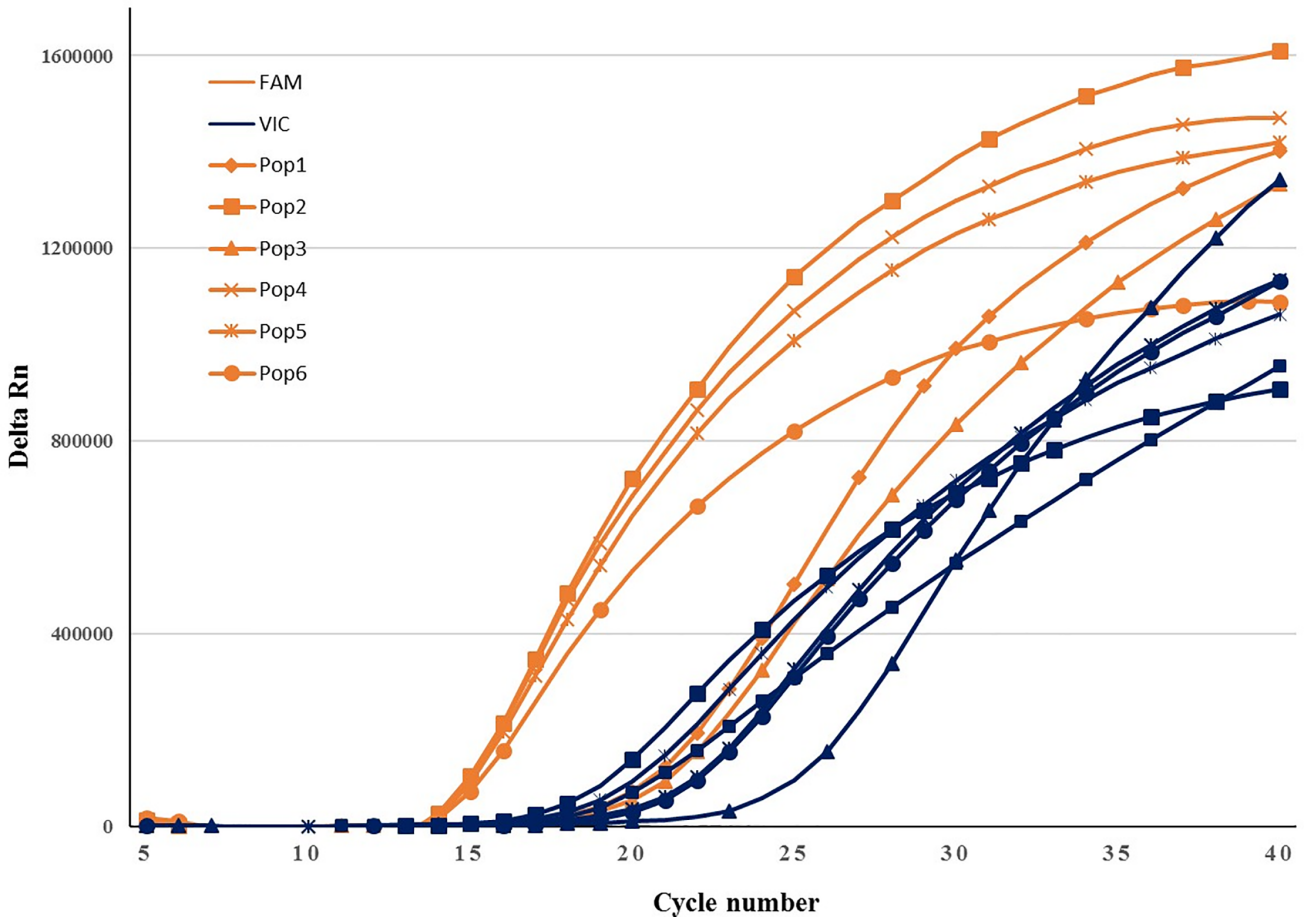
Amplification plot obtained during multiplex real-time PCR on DNA of specimens belonging to different populations of *B*. *zonata* (Numbers 1, 3–5, 10 and 12 in Table 4; Population 1: Konarak, Population2: Minab, Population 3: Rudan, Population 4: Zarabad, Population 5: Tiskupan, Population 6: Chabahar); a) COI (FAM) and b) 18S internal control (VIC).

*B*. *zonata* specimens were successfully identified using the TaqMan probe and primer pair, regardless of their life stages (Fig 1). All of the *B*. *zonata* samples tested with the multiplex real- time PCR generated Cq values for both COI (FAM) and 18S markers (Fig 2; Table 4). Similar ranges of Cq values for the *B*. *zonata* specimens were generated using the COI probe system (FAM) regardless if it is multiplexed (12.37–14.1) or not multiplexed (12.79–15.07) with 18S in the assay, demonstrating that the assay was not negatively affected by multiplexing. The cut-off of the assay was considered 30 cycles in order to decrease the possibility of non-specific binding. Nonetheless, no nonspecific amplification was observed.

### Assay specificity

All 19 non-target tephritid species tested in the multiplex assay generated Cq values for the 18S (VIC) probe ranging between 10.02 and 21.06. None of the non-target species generated a Cq value with the *B*. *zonata* (FAM) probe (Table 4).

### Analytical sensitivity and performance of the assay

**Fig 3:**
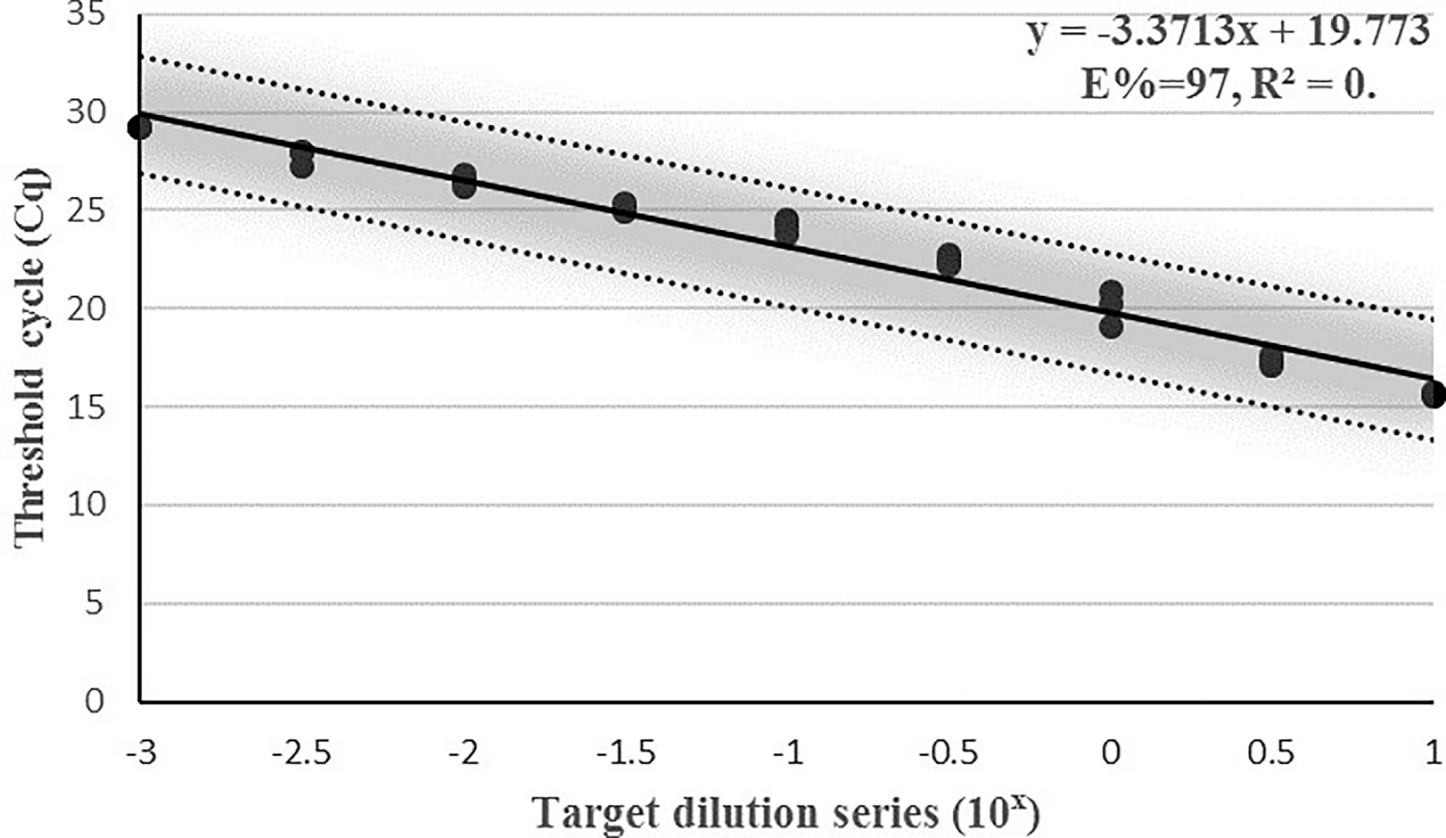
Efficiency of the real-time PCR assay for the identification of *B*. *zonata*. Dilution series were used to create calibration curves for Efficiency calculations. The standard curve built from Cq (threshold cycle) values against the target dilution series (range 1400 pg/μl to 1.4 pg/μl). The 97% confidence interval of the slope is shaded. Based on curve statistics, the assay efficiency is 97.0% and the *r*^*2*^ (fit) = 0.9722.

As expected, with decreasing DNA template concentrations, the Cq values of the reactions increase (Fig 3). The PCR efficiency (97.98%) was within the generally accepted range for an efficient PCR reaction (95–105%) (Fig 3).

The linear dynamic range of the assay extended from 10^1^–10^3^ dilutions of template DNA. The correlation coefficient, *r*^*2*^, of the calibration curve was 0.972. The confidence limits of the linear dynamic range are plotted in Fig 3. High assay reproducibility was demonstrated by low variation in CV% values for each of the samples tested within individual and between different runs (Table 5).

**Table 5:** Repeatability (the intra-run variation of a sample) and reproducibility (inter-run variation of a sample) of the assay, measured as percentage coefficient of variation (%CV).

Sample	Repeatability (Run 1, %CV)	Repeatability (Run 2, %CV)	Reproducibility (Run 1 *vs* Run 2, %CV)
1	0.128	1.743	1.26
2	2.486	1.89	2.36
3	3.48	2.74	3.52
4	0.575	1.444	4.98
5	3.33	1.49	4.54
6	4.50	3.49	4.10

**Fig 4:**
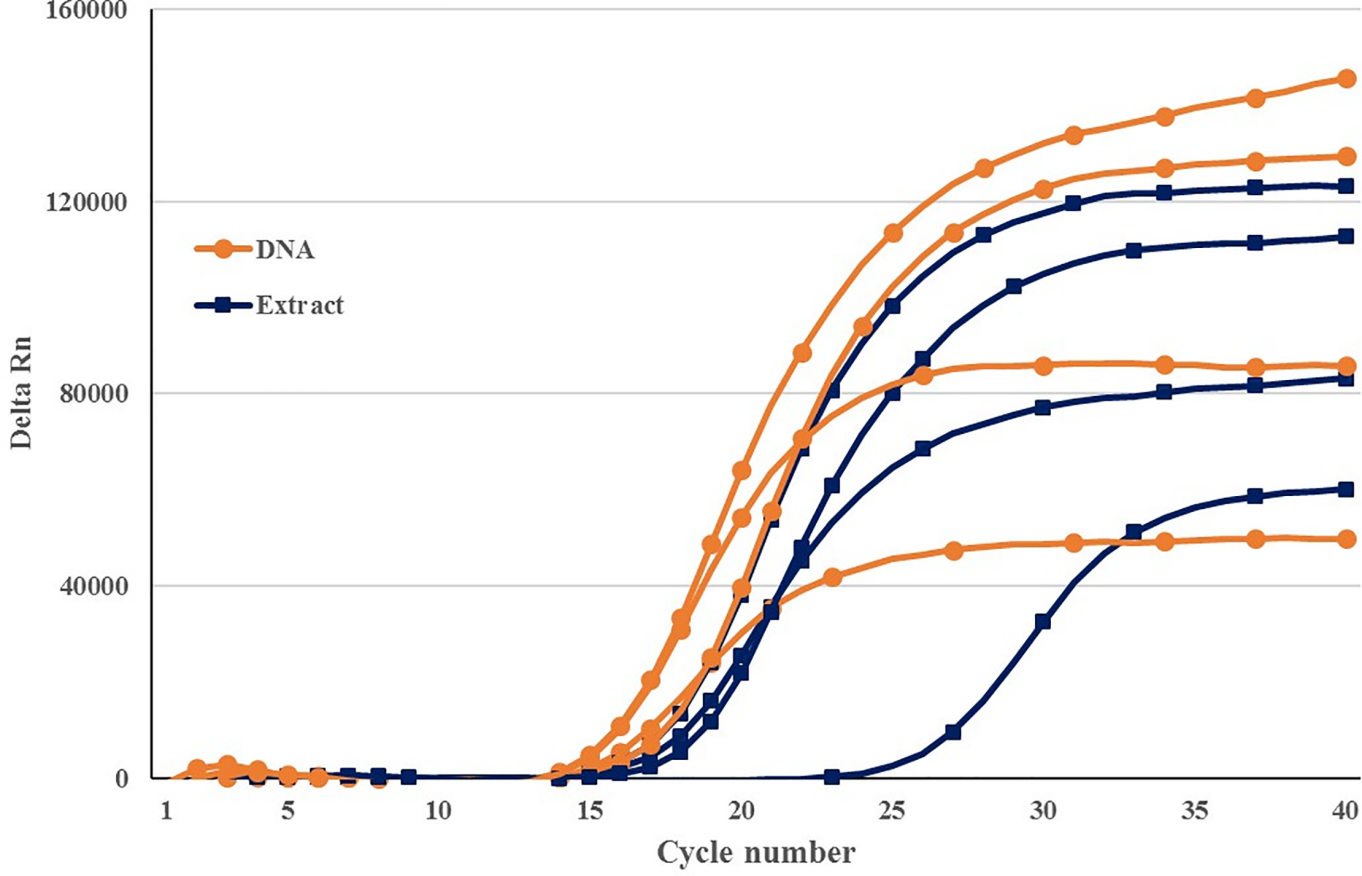
Amplification plot obtained during real-time PCR on DNA of *B*. *zonata* with and without DNA extraction.

By excluding DNA extraction step and utilizing crushed individuals as template, the results did not change and the target *B*. *zonata* samples were amplified successfully within the Cq cut-off 30 cycles (Fig 4).

### Limit of detection

The limit of detection (LOD) of the assay was 1.4 pg of target DNA. The calibration curve (Fig 3) was able to detect all the samples at 1400 pg/μl to 1.4 pg/μl concentration.

### In-silico testing for specificity

Using the “Test with saved primers” tool in Geneious 10.1.3, 1 allowed mismatches on each of the primer/probe sequences was tested, the successful amplification with the primers and probe developed here. Only the four *B*. *zonata* sequences returned a successful match (S1 Appendix).

## Discussion

Border biosecurity depends immensely on the capability of swift, accurate and reliable detection and diagnosis of pests that jeopardize trade. Although various detection methods are currently being utilized to identify intercepted pests, morphology-based tools are often unreliable and time consuming, causing significant delays at the borders. Real-time PCR-based techniques have revolutionized this process in recent years and are increasingly being applied to identify pests around the world [27,41,42]. Nonetheless, species-specific real-time PCR assays are only available for a minority of either economically significant or difficult to identify insect pests including fruit flies–*Bactrocera latifrons* [28], *Bactrocera phillippinensis* and *Bactrocera occipitalis* [29], *B*. *invadens*, *B*. *tryoni*, *C*. *capitata*, *Dirioxa pornia* [41], *B*. *xanthodes* [43]; sap-sucking insects—*Thrips palmi* [22], *Epiphyas postvittana* [23], *Aphis glycines* [26], *Halyomorpha halys* [27]; and *Drosophila suzukii* [11]. Morphological identification of these species is impeded as they are often intercepted as immatures [41]. Likewise, *B*. *zonata*, which is considered as an economically significant tephritid pest, is also strenuous to identify in the immature stages.

In this study, *B*. *zonata* samples were identified accurately using a real-time PCR assay regardless of the developmental stages. By eliminating post-PCR processing, overall identification time was decreased by several hours. Moreover, consistent results were obtained using crushed insects as template, omitting the DNA isolation step, which in turn, results in further time saving. The approximate time needed for performing the TaqMan real-time PCR was significantly shorter compared to that needed for some other relevant methods of identification such as the conventional PCR or the DNA barcoding. Therefore, this technique was less labor-intensive and time-consuming. Moreover, TaqMan probes generate high specificity [44] and could be utilized in any moderately equipped laboratory [27].

Since none of the 19 additional non-target tephritid species included in our study cross-react with the COI probe, it can be concluded that the assay is specific enough to differentiate *B*. *zonata* from either sympatric, closely related species or the species with shared host plants. The results of *in silico* testing with a relatively larger number of additional species further expands the applicability of this assay. Hence, quick and precise identification of the *B*. *zonata* is possible via TaqMan real-time PCR approach.

Swift outcomes along with high specificity and high sensitivity are the three foremost principles which should be noted while applying any diagnostic assay, particularly within the quarantine framework. Although all of these criteria were fulfilled with TaqMan real-time PCR assay used here, saving time is the most remarkable benefit of the technique compared to the traditional morphology-based or conventional PCR-based methods of identification.

An optimal percent of accuracy and reliability (100%) was obtained in the current study in diagnostic and analytical specificity assays. The high efficiency and sensitivity further certify the consistency of the results. Moreover, the immensely low intra-assay and inter-assay variance confirms the feasible application of the assay throughout various laboratories.

In conclusion, the TaqMan qPCR assay used here meets the requirements of quarantine security agencies to screen and decelerate the further spread of this pest in order to facilitate frictionless global trade. This method is also amenable for high-throughput deployment, such as range delimitation, in the event of an incursion into new territory.

## Supporting information

S1 Appendix Alignment of 131 sequences including four *B*. *zonata* sequences to form the in-silico test set using Clustal W implemented in Genefious 10.1.3. (JPG)
