# Supplementary figures and images for: Rapid identification of Bactrocera zonata (Dip.: Tephritidae) using TaqMan real-time PCR assay
Source: PLoS One. 2018 Oct 4;13(10):e0205136. doi: 10.1371/journal.pone.0205136 (PMC6171934; doi:10.1371/journal.pone.0205136)

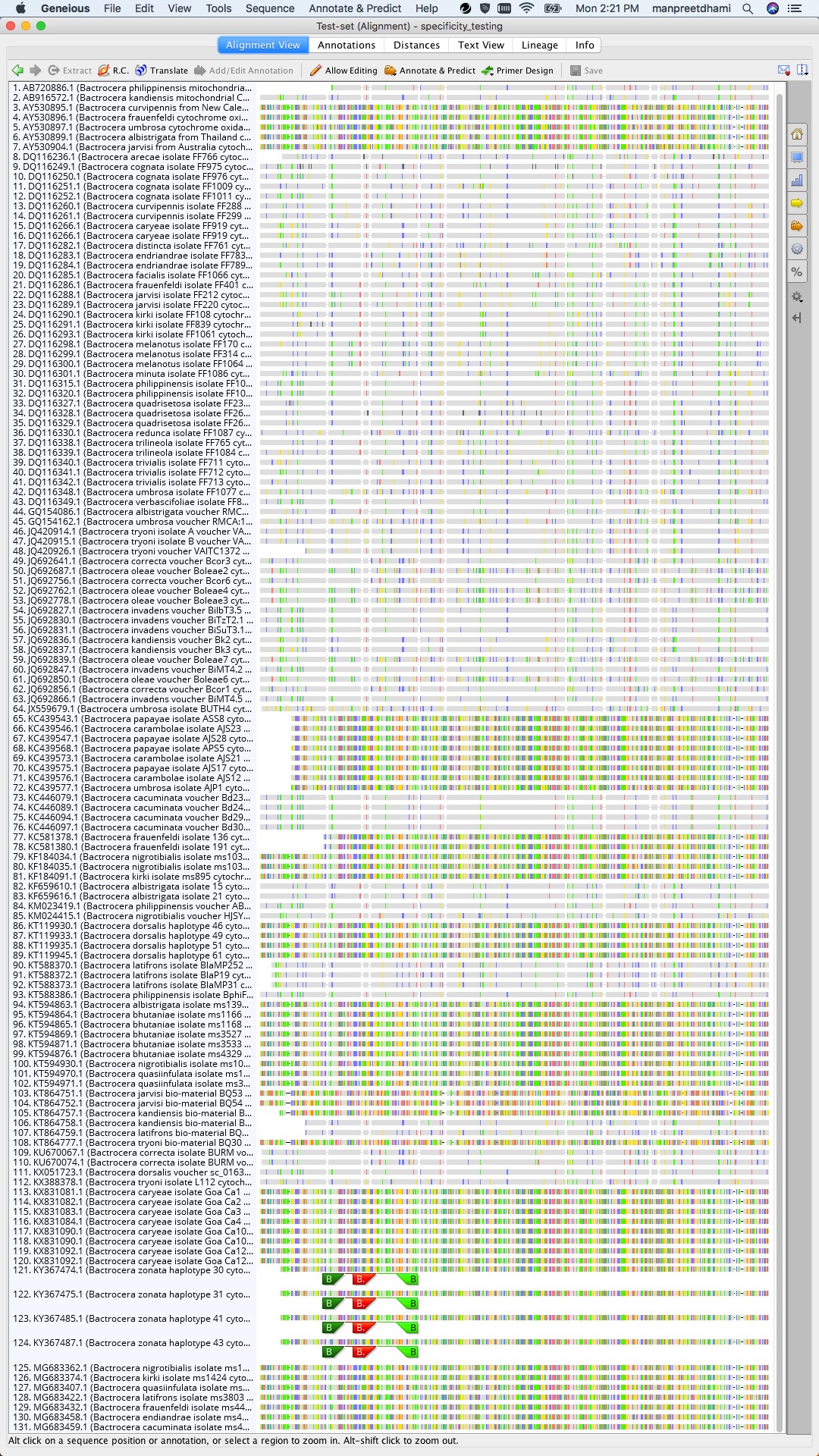

Supplement: S1 Appendix — (JPG) [file pone.0205136.s001.jpg]
